# Stress fibers of the aortic smooth muscle cells in tissues do not align with the principal strain direction during intraluminal pressurization

**DOI:** 10.1007/s10237-021-01427-7

**Published:** 2021-01-30

**Authors:** Shukei Sugita, Naoto Mizuno, Yoshihiro Ujihara, Masanori Nakamura

**Affiliations:** 1 Biomechanics Laboratory, Department of Electrical and Mechanical Engineering, Graduate School of Engineering, Nagoya Institute of Technology, Gokiso-cho, Showa-ku, Nagoya, 466-8555 Japan; 2 Center of Biomedical Physics and Information Technology, Nagoya Institute of Technology, Gokiso-cho, Showa-ku, Nagoya, Japan; 3 Department of Nanopharmaceutical Sciences, Nagoya Institute of Technology, Gokiso-cho, Showa-ku, Nagoya, Japan

**Keywords:** Thoracic aorta, Hypertension, Stress fiber, Mechanotransduction, Principal direction, Photobleaching

## Abstract

Stress fibers (SFs) in cells transmit external forces to cell nuclei, altering the DNA structure, gene expression, and cell activity. To determine whether SFs are involved in mechanosignal transduction upon intraluminal pressure, this study investigated the SF direction in smooth muscle cells (SMCs) in aortic tissue and strain in the SF direction. Aortic tissues were fixed under physiological pressure of 120 mmHg. First, we observed fluorescently labeled SFs using two-photon microscopy. It was revealed that SFs in the same smooth muscle layers were aligned in almost the same direction, and the absolute value of the alignment angle from the circumferential direction was 16.8° ± 5.2° (*n* = 96, mean ± SD). Second, we quantified the strain field in the aortic tissue in reference to photo-bleached markers. It was found in the radial-circumferential plane that the largest strain direction was − 21.3° ± 11.1°, and the zero normal strain direction was 28.1° ± 10.2°. Thus, the SFs in aortic SMCs were not in line with neither the largest strain direction nor the zero strain direction, although their orientation was relatively close to the zero strain direction. These results suggest that SFs in aortic SMCs undergo stretch, but not maximal and transmit the force to nuclei under intraluminal pressure.

**Supplementary information:**

The online version contains supplementary material available at (10.1007/s10237-021-01427-7).

## Introduction

Hypertension is associated with aortic wall thickening, which occurs as homeostasis to keep circumferential stress in the aortic wall constant (Matsumoto and Hayashi 1996a, 1996b). Hypertensive responses in the aortic wall of rats include smooth muscle cell (SMC) hypertrophy (Wiener et al. 1977) and proliferation (Wu et al. 2011) and an increase in ground substances (Matsumoto and Hayashi 1996b), with no change in the number of smooth muscle–rich layers (SMLs) and elastic laminas (ELs). During a cardiac cycle, a pulse pressure in the aortic wall causes SMCs to cyclically circumferential stretch as they align in the circumferential direction (Liu 1998). In vitro studies have shown that cyclic stretch causes SMCs to proliferate (Li et al. 1997; Song et al. 2012) and alter collagen α_1_ messenger RNA (mRNA) expression (Stanley et al. 2000) and fibronectin production rates (Stanley et al. 2000). These results indicate that increased SMC stretch promotes aortic wall hypertrophy in hypertension patients.

Stress fibers (SFs) are cellular mechanosensors that sense environmental forces. SFs work as a force-transmitting and force-focusing molecular “device” and transmit external forces to distant places in cells (Hayakawa et al. 2008; Wang and Suo 2005). Nagayama et al (2011) cut SFs by laser ablation and found that relocation of cell nucleus took place. This means that some of SFs were connected to cell nuclei, and external forces were physically transmitted to the nucleus through SFs. Nagayama et al. also reported that SF cutting also changed DNA distribution within the nuclei (Nagayama et al. 2011). These results offer a hypothesis that external forces transmitted to cell nucleus through SFs lead to nuclear deformation and promote alterations in cellular functions. This hypothesis is supported by various experiments. For instance, compressive forces cause chromatin condensation and cell proliferation (Versaevel et al. 2012). External forces acting on the cell membrane induce chromatin deformation and dihydrofolate reductase upregulation; however, disruption of actin bundles attenuates the transmission of external forces (Tajik et al. 2016). These results show that SFs transmit external forces to the cell nuclei, altering the DNA structure, gene expression, and cell activity.

The magnitude of a force transmitted to cell nuclei might vary depending on the relative direction of SFs to the force applied, and changes in the magnitude of the force transmitted might provoke different cell responses. This hypothesis is derived from two studies. First, patellar tendon fibroblasts cyclically stretched parallel to their major axis in vitro increase the alpha-smooth muscle actin (α-SMA) production rate, while those cyclically stretched perpendicular to their major axis do not (Wang et al. 2005). Second, SFs in SMCs isolated from the aorta basically align in the cellular major axis direction (Nagayama and Matsumoto 2004). Although supportive information has been obtained by a later in vitro study (Kaunas et al. 2006), little is known about cellular deformation and biochemical responses in concern with SF orientations in the in vivo state.

In the aorta in vivo, SFs are not arranged parallel to the major axis of SMCs. Karimi and Milewicz (2016) reported oblique SF alignment across SMCs in the radial-circumferential plane in vivo. However, they did not measure alignment angles of SFs, so how much force is transmitted through SFs in SMCs is unclear.

Aortic tissue deformation under intraluminal pressure is complex. At the cellular scale, aortic tissue is not just stretched in the circumferential direction (Sugita et al. 2020). Strain analysis shows radial-circumferential shear deformation and almost zero radial normal strain with a positive circumferential normal strain (Sugita et al. 2020), indicating that the zero normal strain direction exists in the radial-circumferential plane between the radial and circumferential directions. If SFs align in the zero normal strain direction, SFs in aortic SMCs undergo no stretch under intraluminal pressure.

This study determined whether SFs in aortic SMCs undergo stretch under intraluminal pressure. First, we analyzed the direction of SFs in aortic SMCs. Next, we simultaneously measured the strain of aortic tissue and the SF direction in the radial-circumferential plane under intraluminal pressure.

## Materials and methods

### Outline

We performed two types of experiments. First, to obtain the SF angle in vivo, we fixed aortic samples at a physiological pressure and observed fluorescently labeled SFs. Second, to compare the principal direction of intraluminal pressure-induced strain with the SF orientation angle in the aorta, we added strain markers in the ELs by laser photobleaching to unfixed aortic samples at a low pressure and then fixed the samples at a physiological pressure. Next, we calculated a strain tensor from strain marker positions and compared the principal direction with the orientation angle of fluorescently labeled SFs.

### Sample preparation

We obtained 7–10-week-old Slc:ddy male mice (body weight 30–42 g) from Chubu Kagaku Shizai, Nagoya, Japan. Descending thoracic aortas were obtained, as described previously (Sugita et al. 2020; Sugita and Matsumoto 2017). Briefly, we euthanized the mice in a CO_2_ chamber and put gentian violet dots at 3-mm-interval on the ventral side of the aortic surface as in vivo length markers. Next, we excised the aorta and kept it in Krebs–Henseleit buffer until further experiments. All animal experiments were approved by the institutional review board for animal care of the Nagoya Institute of Technology, Nagoya, Japan and followed the guidelines specified by the *Guide for Animal Experimentation, Nagoya Institute of Technology*.

### Fixation under pressure to measure the SF angle

To fix the excised aortic samples in their in vivo state, we fixed the samples under pressure using a pressure-loading device fabricated in a previous study (Sugita et al, 2020). Briefly, we connected both ends of each excised sample to 24G hypodermic needles (NN-2425R; Terumo, Tokyo, Japan). Next, we stretched the samples in the longitudinal direction up to their in vivo length, fixed the hypodermic needles in a tissue bath, and filled the inside and outside of the aorta with 4% paraformaldehyde (PFA, 168–23,255; FUJIFILM Wako, Osaka, Japan). We then connected one hypodermic needle to a hydrostatic pressure bag and applied 120 mmHg of hydrostatic pressure for 12 h at room temperature (~ 26 °C). Finally, the samples were washed with phosphate-buffered saline (PBS[–]).

### SF staining

To observe SFs in the radial-circumferential (*r* − *θ*) plane, we embedded fixed aortic samples in 3% agar solution (01059–85; Nacalai-Tesque, Kyoto, Japan) for 15 min at 4 °C and then sliced them perpendicular to the longitudinal (*z*) axis into 150-μm-thick section using a DTK-1000 microslicer (Dosaka-EM, Kyoto, Japan).

Next, we placed the sections inside a flow cell made with a 25 × 60 C025601 coverslip (Matsunami, Kishiwada, Japan) and a 18 × 18 C218181 coverslip (Matsunami) glued together with 86-μm-thick NW-10 double-sided tape (Nichiban, Tokyo, Japan). The flow cell, box shape (18 × 10 × 0.15 mm^3^), was filled with 200 × diluted Alexa Fluor 488 phalloidin (A12379; Invitrogen, Waltham, MA, USA) in PBS(–) including 0.2% bovine serum albumin (BSA, 019–23,293; FUJIFILM Wako), incubated for 4 h at room temperature, and washed thrice with PBS(–).

### Two-photon microscopy

We observed elastin autofluorescence of ELs and fluorescently labeled SFs under an Olympus FV1200MPE two-photon laser scanning microscope (Olympus, Tokyo, Japan) equipped with a mode-locked Ti:sapphire laser (pulse width 100 fs, repetition frequency 80 MHz, wavelength 800 nm; MaiThai, Spectra Physics, Mountain View, CA, USA). We observed samples using optical filters (dichroic mirror, 485 nm; band-pass filter for elastin, 420–460 nm; and band-pass filter for SFs, 495–540 nm; FV10-MRV/G, Olympus) and a LUMPLFLN 60XW objective lens (numerical aperture [NA] 1.00; Olympus).

For aortic samples fixed at 120 mmHg and sectioned, the radial-circumferential planes at 15–30 μm depth from the surface were imaged from *z* direction with the *z* interval of 1 μm. For tubular aortic samples pressurized at 15 mmHg to obtain the reference state of strain, the longitudinal-circumferential planes were imaged from the outside of the adventitia with the *r* interval of 1 μm. To compare the SF direction between local positions, we imaged the ventral, dorsal, and lateral sides of the aorta.

### Analysis of the SF direction

**Fig. 1:**
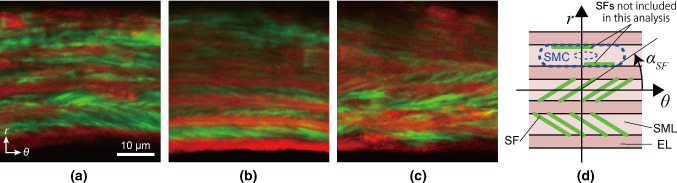
Two-photon images of the aorta labeled with elastic laminas (red) and stress fibers (green) in the radial-circumferential (*r*-*θ*) plane. Images were captured on the **a** ventral, b lateral, and **c** dorsal sides of the thoracic aorta. The sign of the angle was determined, as shown in **d**. SFs that lined near and parallel to ELs are not included in the analysis. Image contrast was adjusted for clear visibility

We analyzed all images using ImageJ v. 1.52p (National Institutes of Health, Bethesda, MD, USA). First, we stacked radial-circumferential plane images of EL and SFs in the *z* direction and implemented the maximum intensity projection in the *z* direction. Next, we defined the circumferential direction on the maximum intensity projection image as the overall direction of ELs, which we determined using the Directionality function (Schindelin et al. 2012). The direction angle between SFs and ELs was measured manually for 3–5 SFs in each SML, and the average angle, *α*_SF_, was acquired; the angle was considered positive for counterclockwise oblique alignment from the circumferential direction, as shown in Fig. 1d. We analyzed only clearly visible SFs, and excluded ones that lied near and parallel to ELs because it is suggested that SFs located away from the nucleus were not physically connected with the nucleus (Nagayama et al. 2011), and thus, they did not seem to transmit force to the nucleus.

### Strain markers

We made strain markers by photobleaching ELs to the samples to simultaneously measure the SF direction and local strain. The detailed process is given (Sugita et al. 2020). Briefly, we pressurized tubular aortic samples at 15 mmHg without fixation and observed autofluorescence of ELs from the outer side of the adventitia in the longitudinal-circumferential plane under the two-photon microscope. Strain markers were produced by local photobleaching of ELs with 3–30% of maximal laser power for ~ 5 min. The laser was irradiated through 60 × objective lens with 50 × optical zoom function. Strain markers were made at all ELs with a 100-μm-interval in the circumferential direction and 40–70-μm-intervals in the longitudinal direction.

### Strain analysis

We determined the strain in reference to strain marker positions at 15 mmHg using isoparametric mapping with a first-order shape function, as described previously (Sugita et al. 2020). Although strain markers made at 15 mmHg were clearly seen at 120 mmHg, their correspondence was difficult to identify. It was because the markers at 15 and 120 mmHg were imaged differently. The markers at 15 mmHg were observed in a tubular sample. The images were taken in the longitudinal-circumferential planes and digitally converted to the radial-circumferential plane images. In contrast, the markers at 120 mmHg were directly observed in the radial-circumferential plane. The samples observed were not tubular, but sectioned perpendicular to the longitudinal direction. Because of this sectioning process, we failed to identify the correspondence of the markers between 15 and 120 mmHg with confidence. Thus, we decided to apply the same reference frame for all samples when calculating strains at 120 mmHg. The reference frame was obtained by longitudinally averaging marker positions at 15 mmHg in each mouse (see Supplementary material S1). We calculated the following strains and directions: circumferential normal strain *ε*_*θθ*_, radial normal strain *ε*_rr_, radial-circumferential shear strain *ε*_r*θ*_, first principal strain *ε*_1_, second principal strain *ε*_2_, and first *α*_1_ and second principal directions *α*_2_ (see Supplementary material S2 in details). The normal strain in the SF direction was calculated as follows:

1 $$\varepsilon_{SF} = \frac{{\varepsilon_{1} + \varepsilon_{2} }}{2} + \frac{{\varepsilon_{1} - \varepsilon_{2} }}{2}\cos 2\left( {\alpha_{SF} - \alpha_{1} } \right).$$

The zero normal strain directions, $\alpha_{{{\text{min}}1}}$ and $\alpha_{{{\text{min}}2}}$, were determined as follows:

2 $$\alpha_{{{\text{min}}1}} = \frac{{180^\circ - \cos^{ - 1} \left( {\frac{{\varepsilon_{1} + \varepsilon_{2} }}{{\varepsilon_{1} - \varepsilon_{2} }}} \right)}}{2} + \alpha_{1}$$

3 $$\alpha_{{{\text{min}}2}} = - \frac{{180^\circ - \cos^{ - 1} \left( {\frac{{\varepsilon_{1} + \varepsilon_{2} }}{{\varepsilon_{1} - \varepsilon_{2} }}} \right)}}{2} + \alpha_{1}$$

From two directions of zero normal strain, we chose the one closer to the circumferential direction and defined it as *α*_min_, because SFs were observed to align closer to the circumferential direction than the radial direction.

4 $$\alpha_{{{\text{min}}}} = \left\{ {\begin{array}{*{20}c} {\alpha_{{{\text{min}}1}} } & {\left( {|\alpha_{{{\text{min}}1}} \left| {\left\langle \right|\alpha_{{{\text{min}}2}} } \right|} \right)} \\ {\alpha_{{{\text{min}}2}} } & {\left( {|\alpha_{{{\text{min}}1}} \left| { \ge } \right|\alpha_{{{\text{min}}2}} |} \right)} \\ \end{array} } \right.$$

### Statistical analysis

Statistical analysis was performed using R software v. 3.6.0 (R core team). Data were shown as the mean ± standard deviation. Absolute values of SF angles biased from zero were evaluated using the *t*-test, and a comparison between more than two groups was tested using the Steel–Dwass test. *P* < 0.05 was considered statistically significant.

## Results

### SF direction

Figure 1 shows typical images of SFs obliquely aligned across SMLs in the radial-circumferential plane. We observed both positive and negative SF angles. SFs in a single SML were aligned in almost the same direction, although some SMLs showed the coexistence of positively and negatively aligned SFs (Supplementary Fig. S3). A comparison of SF directions between two neighboring SMLs showed a directional change of SFs between them (Fig. 1a, bottom two layers, and Fig. 1b, bottom three layers), as described previously (Karimi and Milewicz 2016).

**Fig. 2:**
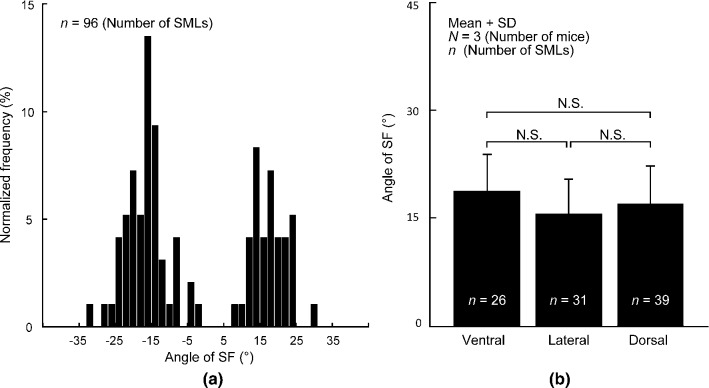
SF direction in the thoracic aorta. **a** Normalized frequency of stress fibers (SFs) oriented from elastic laminas in the radial-circumferential plane. Angles of 0° and ± 90° correspond to circumferential and radial directions, respectively. **b** Absolute value of the SF direction on ventral, lateral, and dorsal sides of the thoracic aorta

Figure 2a shows the frequency distribution of the SF alignment angle. The SF angle obtained from all data was *α*_SF_ =  − 2.6° ± 17.6° (*n* = 96). We found no SFs in the range − 1° ≤ *α*_SF_ < 7°, indicating that SFs are not aligned in the circumferential direction. In addition, no SFs were aligned in the range *α*_SF_ <  − 33° or 31° ≤ *α*_SF_. The frequency distribution showed two distinct peaks, indicating that SFs are aligned in a specific direction. Dividing the distribution into two by the border of 0°, that is, positive and negative data, and fitting the Gaussian distribution to each gave us the mean of *α*_SF_ as − 17.7° (*n* = 57) and 16.0° (*n* = 39) for negative and positive distributions, respectively. Taking the absolute value of *α*_SF_, the mean was |*α*_SF_|= 16.8° ± 5.2°, which is significantly different from the circumferential direction (0°).

Figure 2b shows the absolute value of the SF angle at different circumferential positions: |*α*_SF_| was 18.7° ± 5.2° (*n* = 26) on the ventral, 15.5° ± 4.9° (*n* = 31) in the lateral, and 17.0° ± 5.3° (*n* = 39) in the dorsal side. There was no significant difference between the three groups.

### Strain and SF directions

**Fig. 3:**
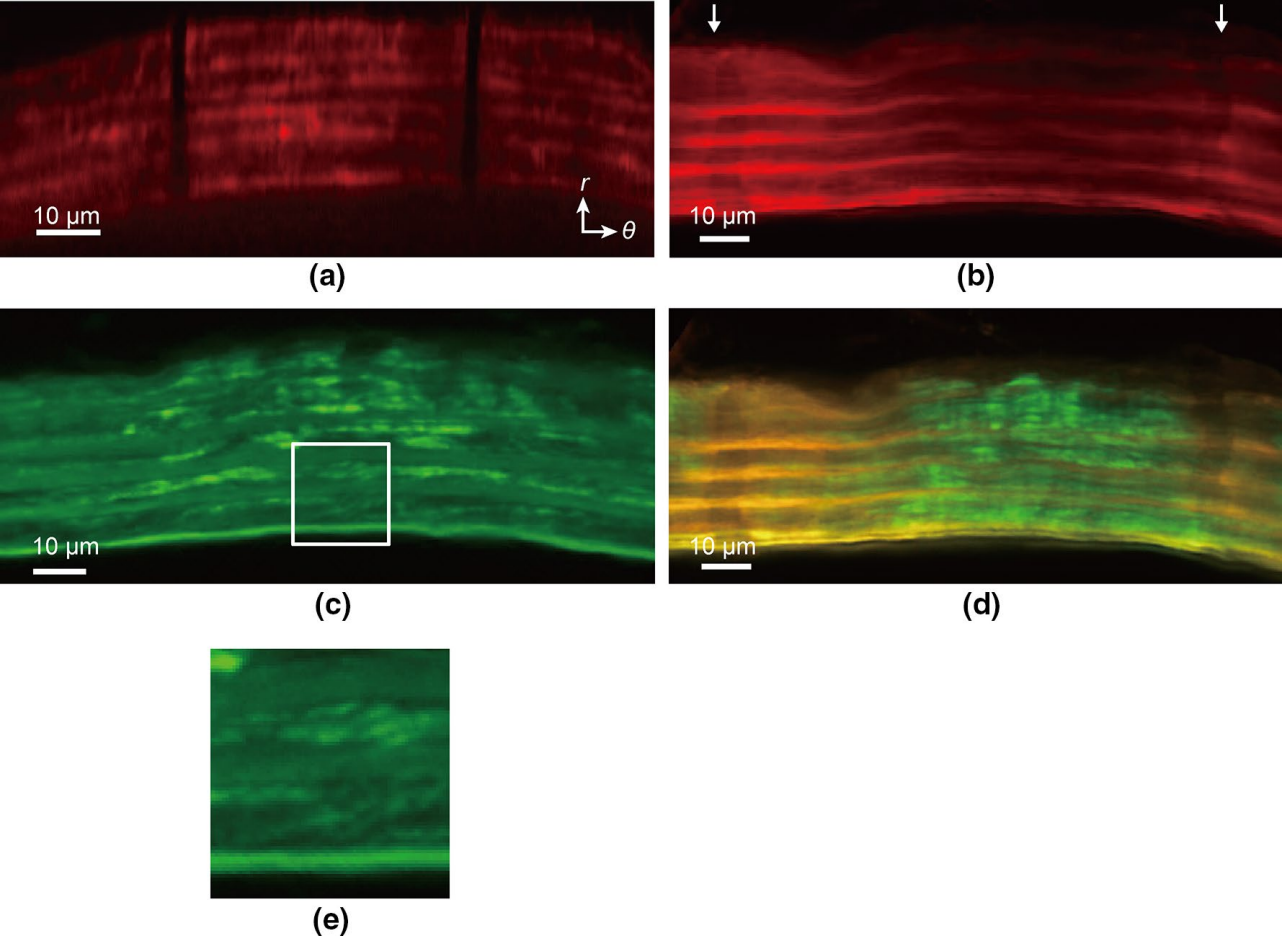
Simultaneous observation of elastic laminas under intraluminal pressure and stress fiber (SF) angle in the radial-circumferential (*r*-*θ*) plane. **a** Images of elastin (red) at 15 mmHg. The image **a** was obtained by digital reslice of the image stack of the longitudinal-circumferential planes using ImageJ. **b** Images of elastin at 120 mmHg. Arrows in **b** show strain markers. **c** SFs (green) at 120 mmHg. The plane of **c** 11 μm closer to the objective lens from the plane of **b** was adopted for clarity of SFs. Note that elastin is also in this image for its autofluorescence. **d** Merged image of elastin **b** and SFs in the same plane at 120 mmHg. **e** Magnified image of the squared area in **c** showing oblique alignment of SFs. Image contrast was adjusted for clear visibility

We tested 9 mice and 87 sliced samples and identified markers in only 9 regions of 4 samples (2 mice). Typical image of ELs and SFs, observed simultaneously, is presented in Fig. 3. Figure 3a shows strain markers produced under the intraluminal pressurization of 15 mmHg. The markers were co-aligned in the radial direction, meaning that circumferential coordinates of the markers were the same in all ELs. The marker positions in this condition were defined as the reference frame to calculate strains at 120 mmHg. When the sample was pressurized at 120 mmHg (Fig. 3b), strain markers were no longer aligned radially. This means that their circumferential coordinates were not the same between the ELs (Fig. 3b and d), and SMLs undergo radial-circumferential shear deformation. Interestingly, SFs that were present in the same SML had almost the same alignment angle (Fig. 3c and e).

**Fig. 4:**
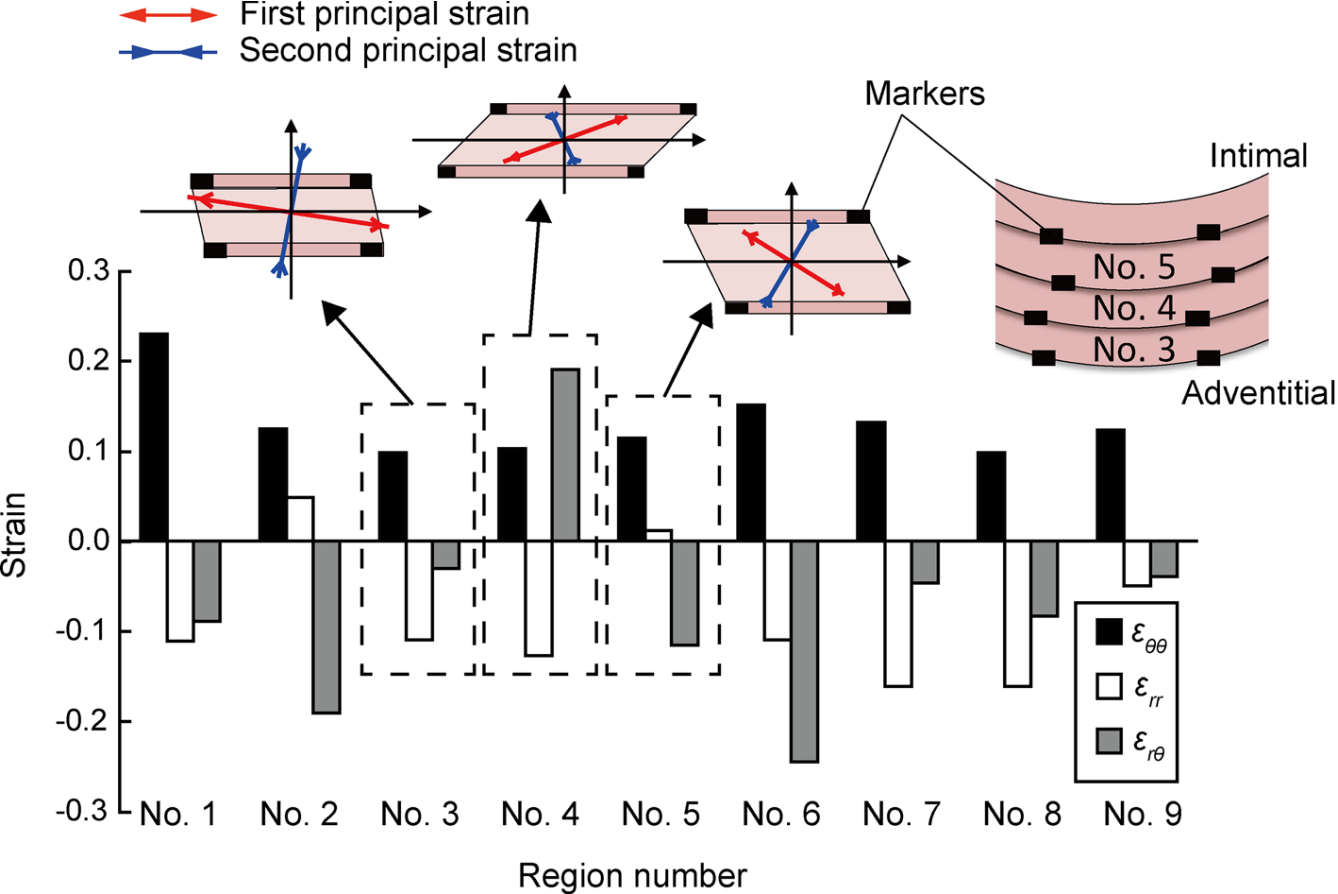
Normal and shear strain in regions 1–9 of smooth muscle–rich layers during pressure from 15 to 120 mmHg. *ε*_*θθ*_, circumferential normal strain; *ε*_rr_, radial normal strain; *ε*_r*θ*_, radial-circumferential shear strain. The schematic deformation state is shown in the upper illustration

Figure 4 shows *ε*_*θθ*_, *ε*_rr_, and *ε*_r*θ*_ in nine regions where we clearly observed SFs. *ε*_*θθ*_ was positive (0.13 ± 0.04), and *ε*_rr_ was either positive or negative (− 0.09 ± 0.07). We also obtained both positive and negative values for *ε*_r*θ*_ (− 0.07 ± 0.12). Regions 3–5 in Fig. 4 are consecutive SMLs. Shear strain was negative in regions 3, 5, and positive in region 4 (the SML sandwiched between regions 3 and 5). These results showed that shear strain directions are different even between adjacent SMLs.

**Fig. 5:**
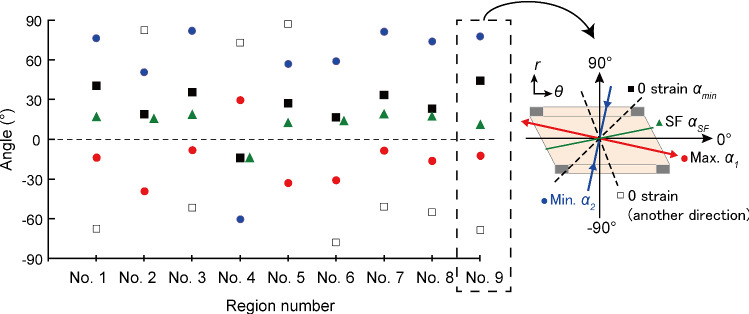
Angles of SFs (green), first principal strain (red), second principal strain (blue), and zero normal strain (black) in regions 1–9 of smooth muscle–rich layers. Angles of 0° and ± 90° correspond to circumferential and radial directions, respectively. A schematic illustration of the angle in region 9 is shown on the right side

Figure 5 shows *α*_1_*, α*_2_, *α*_min_, and *α*_SF_ in each region from the circumferential direction. In all regions, the signs of *α*_1_ and *α*_SF_ were different. The signs of the angles in region 4 were completely opposite to those in other regions. Unifying the direction of shear strains by changing the sign of data in region 4 gave us *α*_SF_ = 15.5° ± 2.7°, *α*_1_ =  − 21.3° ± 11.1°, *α*_2_ = 68.7° ± 11.1°, and *α*_min_ = 28.1° ± 10.2°. These results showed that SFs do not align in the first principal strain direction but that the SF angle is closer to the zero normal strain angle (*α*_SF_ − *α*_min_ = 12.5° ± 10.3°; *n* = 9).

**Fig. 6:**
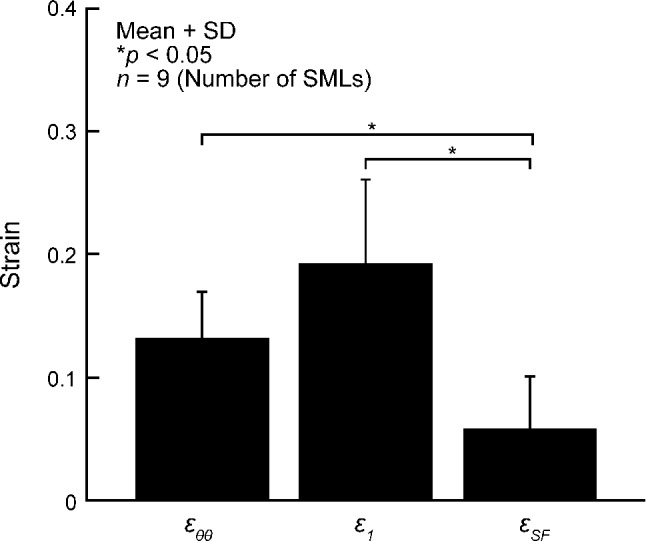
Circumferential normal strain *ε*_*θθ*_, first principal strain *ε*_1_, and strain in the stress fiber direction *ε*_SF_ from 15 to 120 mmHg

Figure 6 compares *ε*_*θθ*_ and *ε*_1_ with *ε*_SF_. *ε*_SF_ was 0.06 ± 0.04, which was significantly smaller than *ε*_*θθ*_ (0.13 ± 0.04) and *ε*_1_ (0.19 ± 0.07). However, *ε*_SF_ was significantly larger than 0.

## Discussion

Previously, we hypothesized that the SFs in in vivo align in the first principal direction *α*_1_ (Sugita et al. 2020) based on following in vitro studies. First, vascular derived cells, embedded in the 3D collagen gel, align parallel to the stretch direction (Foolen et al. 2012; Kanda et al. 1993). Second, SFs in SMCs align in the direction of the cellular major axis (Nagayama and Matsumoto 2004). This study however rejected the hypothesis as demonstrated in Fig. 5. Instead, the data suggested that SFs in SMCs in aortic tissues align such that they avoid undergoing the largest strain during intraluminal pressurization.

When cells are cultured on a 2D silicone rubber sheet, SFs align with the direction perpendicular to the cyclic stretch (Nagayama et al. 2012; Takemasa et al. 1998). In contrast, cells cultured in 3D collagen gel have SFs that align parallel to the cyclic stretch direction (Foolen et al. 2012). These data suggest that SF alignment is determined by a spatial dimension of cellular environment. However, as shown in Fig. 2, in the aorta, the absolute value of the SF angle is approximately 17° from the circumferential direction. This angle is neither the first principal direction nor the zero strain direction. Thus, SF alignment in aortic SMCs obtained in the present study is different compared to 3D collagen gel and 2D silicone rubber sheet.

SFs retain the optimal tension, depending on the stiffness of the environmental substrate, and the direction of SFs is determined by the tension (Tondon and Kaunas 2014). For cells on a 2D silicone rubber sheet, that is*,* a stiff substrate (Young’s modulus ~ 1 MPa), SFs avoid stretch by aligning perpendicular to the direction of the cyclic stretch (Nagayama et al. 2012; Takemasa et al. 1998). The Young’s modulus of aortic tissue is ~ 1 MPa (Learoyd and Taylor 1966), so the elastic modulus of aortic tissue is similar to that of silicone rubber rather than collagen gel. Therefore, the direction of SFs in tissues is determined by the stiffness around SMCs.

The strain magnitude and direction might be affected by the sample preparation in this study, compared with those in unfixed samples subjected to the same loading conditions. The normal circumferential strain at 120 mmHg in this study (*ε*_*θθ*_ = 0.13 ± 0.04) was smaller compared to our previous study (*ε*_*θθ*_ = 0.38) (Sugita et al. 2020). Such a difference was in part attributable to the presence or absence of sample fixation; the present study used fixed samples for simultaneous observation of SF directions and tissue deformation, while the previous study used unfixed samples just for observing tissue deformation (Sugita et al. 2020). According to Fung (1981), elastin do not lose their elasticity completely even if elastin is soaked in fixation agents such as aldehydes for a long period of time (weeks). Because PFA used for fixation in this study is a kind of aldehydes, the sample fixation was considered to be somewhat incomplete. This may have caused release of the strain in the fixed sample, and lead to decreasing *ε*_*θθ*_ in the present study. One may wonder if the incomplete fixation also changed the principal direction *α*_1_. In the previous study, we found that the principal direction *α*_1_ remained almost constant at 40–160 mmHg for unfixed samples (Sugita et al. 2020). Because the strain value (*ε*_*θθ*_ = 0.13 ± 0.04) obtained in the present study is almost equivalent to the one at 40 mmHg in the previous study, we consider that *α*_1_ obtained in the present study would not vary so much from that at 120 mmHg. Therefore, it could be certain that *α*_1_ is far different from *α*_SF_.

Although SFs are not in the largest strain direction, they are also not in line with *α*_min_. *ε*_SF_ is almost one-third of *ε*_1_. A factor that possibly affects *ε*_SF_ is incomplete fixation of elastin, which relaxes SMLs. If circumferential normal strain in samples is released, the aorta at 120 mmHg stretches more in the circumferential direction, resulting in a lower *α*_SF_ from the circumferential direction and higher *ε*_SF_ (Supplementary Fig. S4a and S4b). Also, if circumferential-radial shear strain is released, SFs rotates circumferentially, resulting in a higher *α*_SF_ and lower *ε*_SF_ (Supplementary Fig. S4c and S4d). Thus, the effect of strain release on *α*_SF_ and *ε*_SF_ differs depending on what strain was released. Further investigations are required to determine more precise value of *α*_SF_ and evaluate whether *α*_SF_ equals to *α*_min_ or not.

*α*_SF_ is slightly different from *α*_min_ probably because of the necessity of tension in the actomyosin network to assemble and maintain the SF structure (Kaunas and Deguchi 2011; Polte et al. 2004) and strain (Lu et al. 2008). Cells control tension in SFs through dynamic turnover of constituents and active relaxation. Tension regulation, in turn, enables cells to maintain functional homeostasis. The SF orientation, slightly different from *α*_min_, therefore might give SFs a preferred level of stress or tension.

In this study, we analyzed strain in the circumferential-radial plane. Based on our previous study (Sugita et al. 2020), the normal longitudinal strain is almost zero during pressurization, meaning that no deformation of aortic tissue. This result indicates that SFs neither deform in this direction nor influence the angle of SFs.

Hypertrophic events of the aorta in hypertension patients can be speculated as follows. First, an increase in intraluminal pressure increases the strain in tensed SFs. Next, the SFs mechanically transmit and propagate the strain increase to cell nuclei, for example, by pulling or deforming the nuclei. Third, nucleus deformation restructures the DNA distribution within the nuclei (Nagayama et al. 2011) and stretches chromatin (Tajik et al. 2016), upregulating transcription and cell proliferation (Versaevel et al. 2012).

In a preliminary study, we tried to simultaneously observe SFs and EL markers in the longitudinal-circumferential image stack with intraluminal pressure without specimen fixation. However, SFs in the reconstructed radial-circumferential image were too unclear for their angle to be measured. In the longitudinal-circumferential plane, the authors observed SFs in SMLs of the media through adventitia and several ELs. Consequently, the fluorescent light generated from the SFs was dispersed. In addition, resliced images of the radial-circumferential plane from the longitudinal-circumferential image had less resolution in the radial direction, which significantly degraded the SF image quality and did not allow analysis of SF alignment. Fixation and sectioning improves SF staining, achieving clear images of SFs. However, possible disadvantages are that strain markers cannot be tracked from 15 to 120 mmHg because of sample fixation and finding strain markers in sliced samples is difficult.

It is rare to find fluorescently labeled SFs around strain markers. Because photobleaching is done before fluorescent staining, it is not a result of fluorescence decay from SFs by photobleaching. Laser ablation causes cell apoptosis (Tirlapur et al. 2001), which, in turn, might cause dissociation of actin filaments and deteriorates the SF image quality. Photobleaching does not interfere with the generation of strain (Jayyosi et al. 2014). In addition, the angle of SFs observed simultaneously with ELs is similar to that obtained when only SFs are observed. These data indicate that the effect of photobleaching to make markers is not large enough to change overall results.

## Conclusion

This study measured the SF direction in aortic SMCs and compared it with the strain under intraluminal pressure. SFs are aligned ~ 17° from the circumferential direction in the radial-circumferential plane, the SF direction is completely different from the largest strain direction during pulse pressure changes, and SFs align closer to but not parallel to the zero normal strain direction. Therefore, SFs in aortic SMCs undergo stretch and transmit the force to nuclei under intraluminal pressure.

## Supplementary information

Supplementary file1 (PDF 653kb)

## Data Availability

The datasets generated during and/or analyzed during the current study are available from the corresponding author on reasonable request.
