# Supplementary material for: Stress fibers of the aortic smooth muscle cells in tissues do not align with the principal strain direction during intraluminal pressurization
Source: Biomech Model Mechanobiol. 2021 Jan 30;20(3):1003–11. doi: 10.1007/s10237-021-01427-7 (PMC8154808; doi:10.1007/s10237-021-01427-7)
Supplement: Supplementary file 1 — Supplementary file1 (PDF 653kb) [file 10237_2021_1427_MOESM1_ESM.pdf]

Supplementary material

Title: Stress fibers of the aortic smooth muscle cells in tissues do not align with the principal strain direction during intraluminal pressurization

Journal: *Biomechanics and Modeling in Mechanogiology*

Shukei Sugita[1,2*], Naoto Mizuno[1], Yoshihiro Ujihara[1], and Masanori Nakamura[1,2,3]

Affiliation:

[1] Biomechanics Laboratory, Department of Electrical and Mechanical Engineering, Graduate School of Engineering, Nagoya Institute of Technology, Gokiso-cho, Showa-ku, Nagoya, Japan

[2] Center of Biomedical Physics and Information Technology, Nagoya Institute of Technology, Gokiso-cho, Showa-ku, Nagoya, Japan

[3] Department of Nanopharmaceutical Sciences, Nagoya Institute of Technology, Gokiso-cho, Showa-ku, Nagoya, Japan

To whom correspondence should be addressed:

Shukei Sugita, Ph.D.

Department of Mechanical Engineering, Graduate School of Engineering, Nagoya Institute of Technology

Gokiso-cho, Showa-ku, Nagoya 466-8555, JAPAN

Tel. and Fax: +81 52 735 7125

E-mail: sugita.shukei@nitech.ac.jp

ORCID: 0000-0002-0922-2465

S1. Determination of the original position of strain markers

Although strain markers made at 15 mmHg were clearly seen at 120 mmHg, their correspondence was difficult to identify. Thus, we decided to use the same reference marker positions in each mouse to calculate strains. The reference marker positions were obtained by longitudinally averaging the strain marker positions at 15 mmHg. As seen in Fig. S1a and S1b, there are $N_{EL} \times 2 \times N_z$ strain markers in the $r \times \theta \times z$ directions. For calculating local strain of SML, we selected neighboring four markers (i, j, k, l) (2×2 in the $r \times \theta$ axes), to create a quadrangular grid (Fig. S1c). Their coordinates (r_m, θ_m) ($m = i, j, k, l$) were expressed with radial and circumferential axes. Coordinates of θ_i and θ_l in the circumferential and r_i and r_j in the radial direction were set as 0. In the image at 15 mmHg, the distance between the two strain markers in the circumferential direction in the outermost elastic lamina (EL) was measured (e.g., $L_{\theta 1}$ in the z_1 section in Fig. S1b) and this was repeated for all the pairs of strain markers along the longitudinal direction (e.g., $L_{\theta 1}$–$L_{\theta N_z}$ in the z_1–z_{N_z} sections in the z-axis in Fig. S1b), and their average distance $\bar{\theta}$ was used as coordinates of θ_j and θ_k,

$$\bar{\theta} = \frac{1}{N_z} \sum_{q=1}^{N_z} L_{\theta q}. \quad \text{(S1.1)}$$

To determine the radial direction coordinates, the distance between the strain markers in innermost and outermost layers was measured for two pairs of strain markers in the r-θ plane (e.g., L_{r1} and L_{r2} in the z_1 section in Fig. S1b). This measurement was performed in all r-θ planes along the longitudinal direction (e.g., L_{r1}– L_{r2N_z} in the z_1–z_{N_z} sections in Fig. S1b), and the average of all distances was divided by the number of smooth muscle–rich layers (SMLs) N_{SML},

$$\bar{r} = \frac{1}{2N_z N_{SML}} \sum_{q=1}^{2N_z} L_{rq} \quad \text{(S1.2)}$$

This averaged distance $\bar{r}$ between two adjacent ELs was used to determine the radial direction coordinates r_l and r_k.

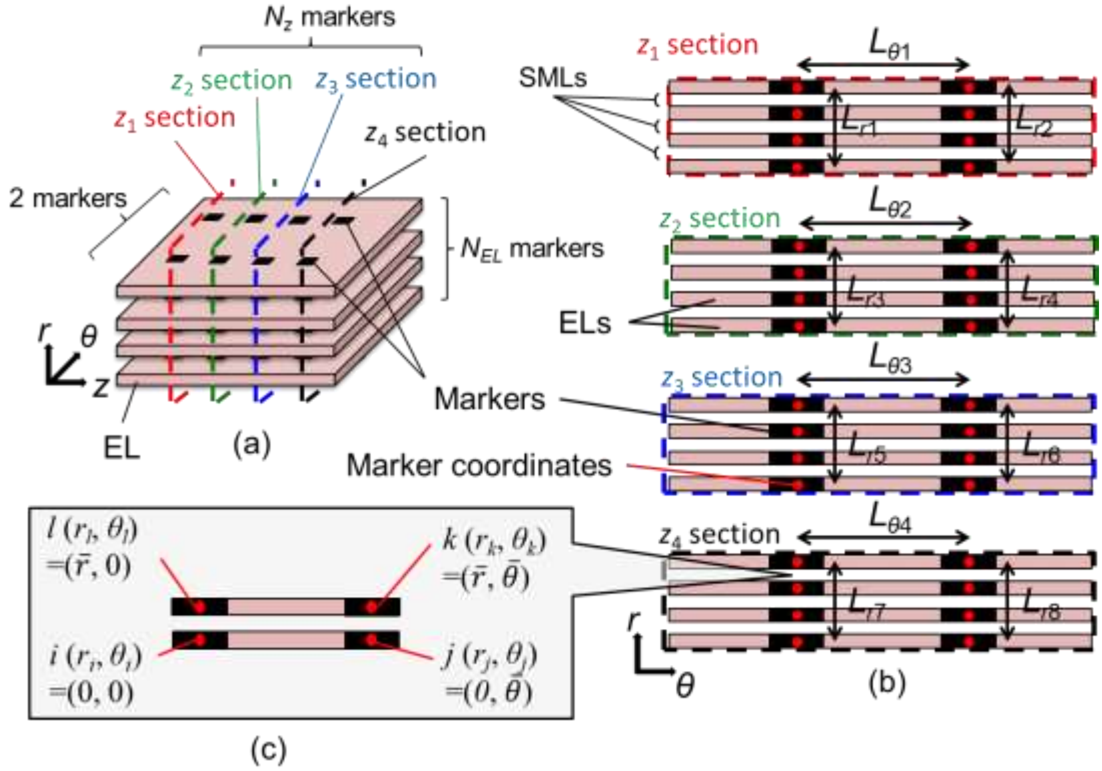

Supplementary Fig. S1 Schematic illustration of strain marker position in elastic laminas and calculated distances between strain markers for strain calculation.

S2. Strain calculation (Sugita et al., 2020)

Strains were calculated in radial-circumferential plane using isoparametric mapping as described in Sugita et al. (2020).

Four nodes i, j, k, and l were selected, and coordinates of the nodes (r_n, θ_n) and displacements (u_n, v_n) of nodes ($n = i, j, k$, and l) between 15 and 120 mmHg pressure levels were measured. A quadrangle element made by the four nodes was mapped to the square which has nodes at (± 1, ± 1) in the ξ-η plane. Quantities including coordinates (r, θ) and its displacement (u, v) in the quadrangle element are written as:

$$\varphi(\xi,\ \eta) = N_i\varphi_i + N_j\varphi_j + N_k\varphi_k + N_l\varphi_l \tag{S2.1}$$

where $\varphi = r$, θ, u, and v, and shape functions N_i, N_j, N_k, and N_l are given by

$$\left.\begin{matrix} N_i = \frac{1}{4}(1-\xi)(1-\eta) & N_j = \frac{1}{4}(1+\xi)(1-\eta) \\ N_k = \frac{1}{4}(1+\xi)(1+\eta) & N_l = \frac{1}{4}(1-\xi)(1+\eta) \end{matrix}\right\}. \tag{S2.2}$$

Normal strains ε_{rr} in the r-direction and $\varepsilon_{\theta\theta}$ in the θ-direction and shear strain in the θ-direction at the surface perpendicular to the r–direction $\varepsilon_{r\theta}$ in the r-θ plane are defined as:

$$\begin{Bmatrix} \varepsilon_{rr} \\ \varepsilon_{\theta\theta} \\ \varepsilon_{r\theta} \end{Bmatrix} = \begin{Bmatrix} \dfrac{\partial u}{\partial r} \\ \dfrac{\partial v}{\partial \theta} \\ \dfrac{1}{2}\left(\dfrac{\partial u}{\partial \theta} + \dfrac{\partial v}{\partial r}\right) \end{Bmatrix}. \tag{S2.3}$$

Strain components in Eq. (S2.3) are obtained by

$$\begin{bmatrix} \dfrac{\partial \kappa}{\partial r} \\ \dfrac{\partial \kappa}{\partial \theta} \end{bmatrix} = \begin{bmatrix} \dfrac{\partial r}{\partial \xi} & \dfrac{\partial \theta}{\partial \xi} \\ \dfrac{\partial r}{\partial \eta} & \dfrac{\partial \theta}{\partial \eta} \end{bmatrix}^{-1} \begin{bmatrix} \dfrac{\partial \kappa}{\partial \xi} \\ \dfrac{\partial \kappa}{\partial \eta} \end{bmatrix}, \tag{S2.4}$$

where $\kappa = u$ and v. From Eqs. (S2.1) to (S2.4), strains ($\varepsilon_{\theta\theta}$, ε_{rr}, $\varepsilon_{r\theta}$) were calculated. In evaluating tissue deformation, strains at (ξ, η) = (0, 0) were used as representatives.

The first and the second principal strains ε_1, ε_2, and the angle of the first principal direction from the circumferential direction α_1 in the radial-circumferential plane were calculated from $\varepsilon_{\theta\theta}$, ε_{rr}, and $\varepsilon_{r\theta}$ as:

$$\begin{Bmatrix} \varepsilon_1 \\ \varepsilon_2 \\ \alpha_1 \end{Bmatrix} = \begin{Bmatrix} \frac{\varepsilon_{rr}+\varepsilon_{\theta\theta}}{2} + \sqrt{\left(\frac{\varepsilon_{rr}-\varepsilon_{\theta\theta}}{2}\right)^2 + {\varepsilon_{r\theta}}^2} \\ \frac{\varepsilon_{rr}+\varepsilon_{\theta\theta}}{2} - \sqrt{\left(\frac{\varepsilon_{rr}-\varepsilon_{\theta\theta}}{2}\right)^2 + {\varepsilon_{r\theta}}^2} \\ \frac{1}{2}\arctan\frac{2\varepsilon_{r\theta}}{\varepsilon_{rr}-\varepsilon_{\theta\theta}} \end{Bmatrix}. \qquad \text{(S2.5)}$$

Reference:

Sugita S, Kato M, Wataru F, Nakamura M (2020) Three-dimensional analysis of the thoracic aorta microscopic deformation during intraluminal pressurization Biomech Model Mechanobiol 19:147-157 doi:10.1007/s10237-019-01201-w

S3. Rare case to have stress fibers (SF) with opposite rotational angles within one SML

It is rare to see both positive and negative SF angles in one SML. Supplementary Fig. S3 shows such a case. In the second SML from the intimal side (bottom left), SFs in the left were aligned in the negative direction, while those in the right were aligned in the positive direction.

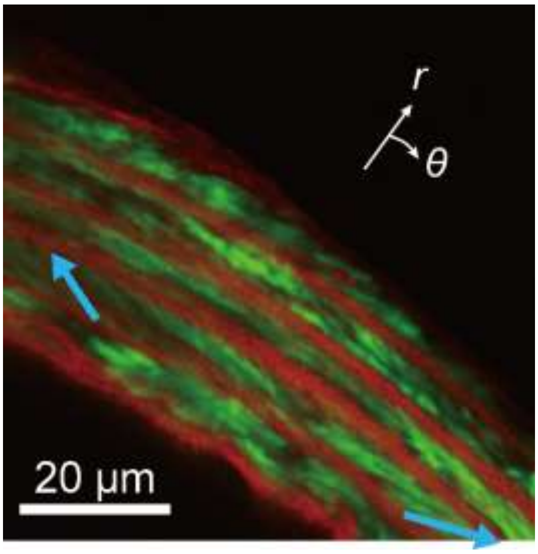

Supplementary Fig. S3 Example of an smooth muscle–rich layer showing the coexistence of positively and negatively aligned stress fibers (SFs). Autofluorescence of elastin (red) and fluorescently labeled SFs (green) were observed. The two blue arrows show SF directions.

S4. The possible effects of strain releasing on the stress fiber angle α_{SF}

The direction of stress fiber (SF) was measured in the specimen fixed with paraformaldehyde. However, incomplete fixation might have released the strain in the specimen. Here, we qualitatively consider the effect of releasing the strain on the direction of SF angle α_{SF} as shown in Fig. S4. If circumferential normal strain is released (Fig. S4a), the actual α_{SF} at 120 mmHg *in vivo* state decreases (Fig. S4b). If the radial-circumferential shear strain is released in (Fig. S4c), the actual α_{SF} at 120 mmHg *in vivo* state increases (Fig. S4d).

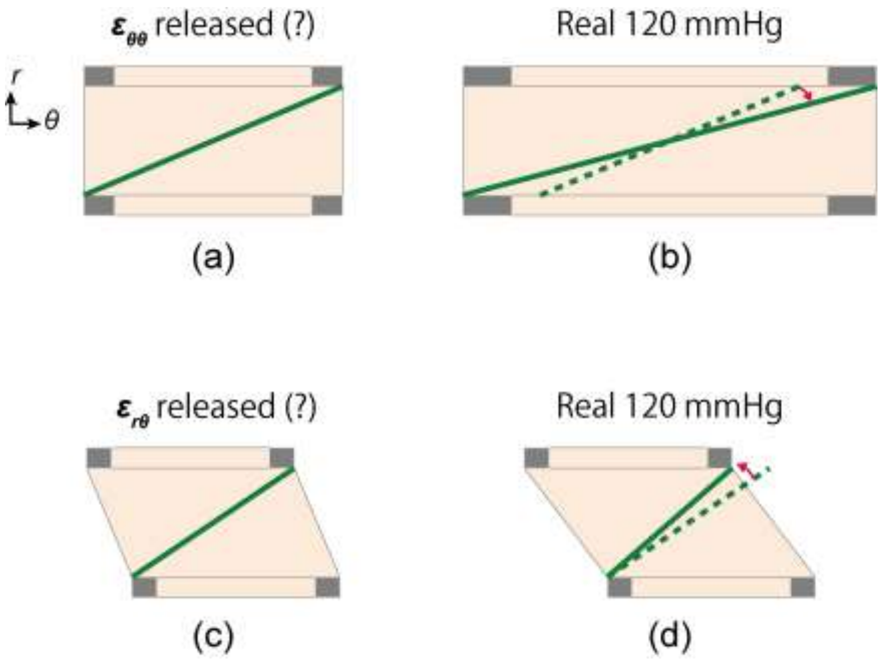

Supplementary Fig. S4 A schematic illustration of the effects of releasing strain on the stress fiber (SF) angle α_{SF}.
